# Left atrial scar burden determined by delayed enhancement cardiac magnetic resonance at post radiofrequency ablation: association with atrial fibrillation recurrence

**DOI:** 10.1186/1532-429X-14-S1-P204

**Published:** 2012-02-01

**Authors:** Gerd Brunner, Lucien Abboud, Kamran A Shaikh, Amish S Dave, Joel Morrisett, William A Zoghbi, Miguel Valderrábano, Dipan J Shah

**Affiliations:** 1 Section of Atherosclerosis and Vascular Medicine, Department of Medicine, Baylor College of Medicine, Houston, TX, USA; 2 The Methodist DeBakey Heart & Vascular Center, Houston, TX, USA

## Background

Left atrial (LA) radiofrequency (RF) ablation has become routine treatment for atrial fibrillation (AF) but still suffers from AF recurrence requiring a repeat procedure. LA-RF ablation success rates vary between 53% and 85%. Delayed-enhancement Cardiac Magnetic Resonance (DE-CMR) can be used to noninvasively visualize LA hyperenhancement (scar). We have utilized DE-CMR to quantify LA scar extent post LA-RF-ablation and related this measure to AF recurrence.

## Methods

Twenty-seven patients (62.0±11.1 years, 20 males) with paroxysmal and chronic AF underwent LA-RF-ablation and subsequent DE-CMR, an average of 260.7±314.7 days post procedure. The DE-CMR procedure was performed utilizing a navigated 3D inversion recovery gradient echo sequence (Siemens 1.5T Avanto or 3.0T Verio) approximately 15 minutes after administration of 0.2 mmol/kg Diethylenetriaminepentaacetic Acid−Gadolinium (DTPA-Gd, Magnevist, Berlex Laboratories, Wayne, NJ). All scans were electrocardiographically (ECG)-gated and acquired during a 150 ms window in mid-diastole with navigator-gating and fat suppression. We have developed an image analysis method and graphical user interface to semi-automatically quantify hyperenhanced regions in the LA wall (scar). LA scar was quantified by a single experienced observer blinded to patient data. LA-scar measurements were normalized by LA size. The intra-class correlation coefficient (ICC) was used to assess intra-observer variability of 4 randomly selected scans which were re-read one week later. Variables were tested for normality with the Shapiro-Wilk test and a p-value<0.05 was considered statistically significant (all tests were 2-sided). All patients provided informed consent.

## Results

The DE-CMR scans were performed 260.7±314.7 days after the initial LA-RF- ablation procedure (Figure). AF recurrence was noted to occur in 13 (48%) patients whereas 14 (52%) patients demonstrated no AF recurrence. There was a trend toward a larger LA-volume in the AF-recurrence group (128.49±44.0 ml vs. 96.0±38.5 ml; p=0.06, see Table 1). Left ventricle ejection fractions (LVEF) were smaller in the AF-recurrence group but the difference was not statistically significant (58.93±12.1% vs. 64.85±6.2.1%, p=0.092). Average analysis time per scan was 14.5±7 min and intra-observer variability was excellent (ICC=0.99). LA-scar was normally distributed (p=0.151). Average LA scar extent, quantified in post LA-RF-ablation DE-CMR scans, was significantly larger in recurrence-free AF patients (16.56±5.3 cm2) when compared with individuals with AF-recurrence (11.40±7.6 cm2; p=0.036). The results indicate that there is a significant inverse relationship between LA-scar burden and AF-recurrence.

**Table 1:** LA-scar quantification in AF patients.

Variable	**AF-Recurrence** [N=13, mean, std]	**AF-Free** [N=14, mean, std]	P-value
LA-Volume [mL]	128.49 ± 44.0	96.0 ± 38.5	0.06
LVEF [%]	58.93 ± 12.1	64.85 ± 6.2.1	0.092
LA-scar [cm^2^]	11.40 ± 7.6	16.56 ± 5.25	**0.036**
Age [years]	61.67 ± 9.3	62.23 ± 12.8	0.891
Gender [no. males]	10	10	-

LA= left atrium; LA scar (hyperenhanced area) was normalized by LA volume. N= number of patients; Std=standard deviation; RF= radio frequency; LVEF: left ventricle ejection fraction; AF: atrial fibrillation.

## Conclusions

LA scar extent can be reproducibly quantified with DE-CMR; and a lower scar burden post LA-RF-ablation is associated with AF recurrence.

## Funding

This work was supported in part by NIH grant T32HL07812.

**Figure 1:**
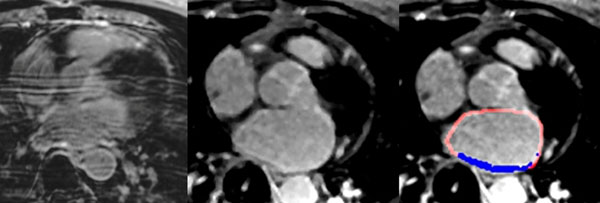
DE-CMR images of the left atrium (LA) obtained with a Siemens 1.5T Avanto (left and middle panels). The right panel shows the result of the semi-automated LA scar segmentation for the center panel. The left atrium is indicated by the red contour and the blue area highlights hyperenhanced regions (scar).

